# Ethylene and the responses of plants to phosphate deficiency

**DOI:** 10.1093/aobpla/plt013

**Published:** 2013-02-26

**Authors:** Marissa Roldan, Phuong Dinh, Susanna Leung, Michael T. McManus

**Affiliations:** 1 Institute of Molecular Biosciences, Massey University, Private Bag 11-222, Palmerston North, New Zealand; 2 Present address: AgResearch Grasslands, Private Bag 11008, Palmerston North, New Zealand; 3 Present address: Department of Plant Pathology, Washington State University, Pullman, WA, USA

**Keywords:** Auxin, ethylene biosynthesis, hormone crosstalk, hormone sensitivity, phosphate supply, root system architecture

## Abstract

The consideration as to how plants uptake and transport phosphorus (P) is of significant agronomic and economic importance, in part driven by finite reserves of rock phosphate. Our understanding of these mechanisms has been greatly advanced, particularly with respect to the responses of plants to P deficiency and the genetic dissection of the signalling involved. Further, the realization that there are two tiers of transcriptional responses, the local, in which inorganic P (Pi) acts as an external signal independent of the endogenous P level, and the systemic involving root–shoot signalling, has now added a dimension of both clarity and complexity. Notwithstanding, it is now clear that the hormone ethylene plays a key role in mediating both levels of responses. This review, therefore, covers the role of ethylene in terms of mediating responses to P deficiency. The evidence that Pi supply regulates ethylene biosynthesis and sensitivity, and that this, in turn, regulates changes in root system architecture and in Pi-deprivation responses is examined here. While ethylene is the focus, the key interactions with auxin are also assessed, but interactions with the other hormone groups, which have recently been reviewed, are not covered. The emerging view that ethylene is a multi-faceted hormone in terms of mediating responses to P deficiency invites the dissection of the transcriptional cues that mediate changes in ethylene biosynthesis and/or sensitivity. Knowledge of the nature of such cues will subsequently reveal more of the underpinning interactions that govern P responses and provide avenues for the production of germplasm with an improved phosphate use efficiency.

## Abstract (precis)

This review considers the evidence that ethylene biosynthesis is up-regulated by locally-generated signals in response to a change in external P supply, where the hormone then mediates, with auxin, changes in root system architecture. Subsequent changes in endogenous P evoke systemic responses whereby ethylene again is important in inducing some of the key signature changes observed in P-deprived tissues (eg. phosphate transporter and acid phosphatase up-regulation).

## Introduction

The mechanisms by which plants access sufficient phosphate (P) from the soil are of intense agronomic and academic interest. The chemical form of phosphorus P taken up by plants, orthophosphate (H_2_PO_4_^−^; designated as Pi), represents only a small fraction of available (free) P in soils and is generally immobile. However, as a macro-element, relatively significant quantities are required for plant function, and measurement of the Pi content of the aerial and root tissue of plants, when compared with the level in the surrounding soil, suggests that efficient uptake and transport systems do operate in plants. The array of Pi transporters, and their expression, has been reviewed relatively recently (Raghothama and Karthikeyan 2005; Nussaume *et al.* 2011), and the determination of the key role of the PHO1 protein has provided significant insight into how plants partition the essential element, in *Arabidopsis thaliana* at least (Chiou and Lin 2011).

Further insight into the mechanisms by which plants uptake and partition phosphate, particularly in terms of changes in root growth dynamics and function, occurs via approaches where phosphate is limiting. In particular, understanding the mechanisms and controls regulating the observed changes in root system architecture (RSA) is an active research field inviting speculation as to how alterations in the levels of both endogenous and exogenous Pi can influence such fundamental (and rapid) changes in growth. In this review, we focus on one aspect of this control: the interaction of Pi level and ethylene biosynthesis and signalling. We examine the emerging view that ethylene may play a role in both modulating RSA and regulating some of the signature cellular changes that accompany the responses of plants to low Pi supply. In terms of RSA, our focus is on changes to primary and lateral root growth. However, it is clear too that root hairs play an important role in Pi acquisition, and while the importance of these structures has been reviewed recently (Peret *et al.* 2011), they will not be considered here. In our examination of the role of ethylene, we will look (briefly) at the role of auxin, but beyond that, and while not wishing to undermine the importance of such interactions, we will not consider the interactions with other hormones in any detail. However, the reader is referred to more recent reviews of this topic (Fukaki and Tasaka 2009; Chiou and Lin 2011). Likewise, there is the intriguing Fe/Pi interaction that is emerging (Ward *et al.* 2008), and which has been reviewed more recently (Abel 2011), and it is also apparent that ethylene signalling is fundamental in terms of plant responses to low-potassium conditions (Kim *et al.* 2012). However, in the space available, we focus on the Pi and ethylene interaction.

## Changes in Phosphate Supply Can Influence RSA

The observation that Pi supply can regulate root architecture arose from early considerations that the subtleties of root structure contribute largely to the efficiency of nutrient uptake, particularly for the immobile elements (Baldwin 1975). Subsequently, changes in RSA (compensatory growth) were observed in barley (Drew 1975; Drew and Saker 1978), and in bean and other legumes (Bonser *et al.* 1996; Borch *et al.* 1999). A summary of changes to the root architecture in bean suggests a root system that undergoes growth responses to maximize top soil foraging, but in tandem with a reduction in the metabolic cost of such soil mining (Lynch and Brown 2001).

More recently, studies have inevitably changed to the model plant species *Arabidopsis.* In addition to the well-known array of genetic resources available, this species has a further advantage for use when dissecting the influence of Pi supply on root growth in that it does not form mycorrhizal associations, so making separation of *in planta* observations more apparent. In comparison with bean, *Arabidopsis* also has a less complex root system when grown from seed over, typically, 14-day time courses. Initial studies using the accession Columbia revealed a signature response to low Pi supply comprising a decreased growth rate of the primary root (PR), in part mediated by a reduction in elongation of cells at the root cap, and an increase in mean lateral root (LR), length (Williamson *et al.* 2001). With the decrease in PR length, LR density thus increased and the distance between the root tip and the first emerged LR also decreased in response to the low Pi treatment (Williamson *et al.* 2001). The decrease in PR length and an increase in LR density in response to low Pi supply were confirmed in Columbia seedlings by López-Bucio *et al.* (2002), with an increase in LR number also contributing. Interestingly, these authors also applied a range of P concentrations and noted a biphasic rather than linear response to P added, particularly in terms of PR length and LR density, perhaps indicating the operation of a ‘threshold’ concentration in terms of the controlling signalling. That these changes represent a re-allocation of resources in *Arabidopsis* was demonstrated by Al-Ghazi *et al.* (2003), who showed that the total root length is unchanged (at least over the period measured), and so with the elongation of the PR decreasing, an increase in LR length is apparent. These authors thus summarized a sequence of changes in RSA for *Arabidopsis* in response to low Pi that involves a decrease in PR elongation, an increase in LR elongation rate, a decrease in the initiation of laterals and an increase in the growth duration of LRs (Al-Ghazi *et al.* 2003).

With these morphological changes in response to low P established in *Arabidopsis*, focus then shifted to understanding the mechanisms by which Pi supply induces these modifications. Nacry *et al.* (2005) showed, using a *CycB1::uidA* reporter construct, that the LR stimulation was mediated *via* the activation of existing primordia, while the initiation of new primordia was repressed in roots exposed to low Pi supply. The decrease in PR elongation involved a shift from indeterminate to determinate growth, with the progressive loss of meristematic cells (Sanchez-Calderon *et al.* 2005). So, how does a root sense a change in Pi supply?

In earlier work, Linkohr *et al.* (2002) were able to show in elegant experiments with nutrient patches that the low P phenotype could be induced or repressed depending on the media through which the root was growing. Indeed, Pi sensing (to trigger reduction of PR elongation) was shown to be resident in the root cap and, significantly, independent of the Pi content of the root (Svistoonoff *et al.* 2007). The emergence of the concept of Pi as a signal had been proposed earlier by Ticconi and colleagues when they showed that application of the phosphate analogue, phosphite, could repress a range of P-starvation responses including transcriptional changes in P-starved seedlings of *Arabidopsis* (Ticconi *et al.* 2001).

In terms of transduction of the Pi signal in *Arabidopsis*, Ticconi *et al.* (2004) identified the *pdr2* locus that is disrupted in Pi sensing, but importantly application of phosphite was able to rescue the mutant phenotype demonstrating that PDR2 was involved in sensing the external Pi availability. Subsequent characterization has determined that PDR2 interacts with LOW PHOSPHATE ROOT 1 (LPR1) in *Arabidopsis*, a regulator previously identified by Svistoonoff *et al.* (2007) as being important in the root tip Pi response (Ticconi *et al.* 2009). The mechanism so revealed suggests that PDR2, in *Arabidopsis* at least, is required for the correct expression of SCARECROW (SCR), a regulator of stem cell maintenance in roots, and thus provides a signalling pathway in which local Pi sensing can lead to the switch from indeterminate to determinate growth in the PR tip as previously reported by Sanchez-Calderon *et al.* (2005).

This role for Pi as a signal was extended in terms of documenting transcriptional changes accompanying local Pi sensing. Using split-root approaches, Thibaud *et al.* (2010) were able to show in *Arabidopsis* that a (low) external Pi, irrespective of internal Pi levels in the root, could influence the transcription of a cluster of genes, designated the locally regulated genes, while a low internal Pi level influenced the transcription of another cluster, designated the systemically regulated genes. Of the genes analysed, the majority were either locally induced (73%) or repressed (68%) and these genes tended to encode proteins involved in hormone biosynthesis and signalling and in stress responses. The systemically induced genes were involved in Pi uptake, mobilization and recycling, suggesting that the changes in external Pi perceived by the plant roots initially elicit a general stress response to perhaps sensitize the plant to any subsequent longer-term nutrient stress. Interestingly, while internal Pi was essential to evoke the systemic response, reducing the flow of Pi into the root did not, suggesting that a Pi-independent signalling system is operating, such as the *PHO2* mRNA regulator, mir399, which is important in coordinating the response. Further, Thibaud *et al.* (2010) were also able to show that the systemically induced genes were marked by the possession of the PiBS motif, a target for the PHR1 transcription factor, while repressed genes do not contain the motif. While *PHR1* itself is only marginally induced by Pi starvation, mutation of the P1BS site impairs P responsiveness, but does not influence responses to other stresses (e.g. wounding) (Bustos *et al.* 2010). The dissection of such internal signalling events in *Arabidopsis*, particularly with respect to shoot-to-root communication, including the key roles of sucrose and MiR399, has been well reviewed recently (Nilsson *et al.* 2010; Yang and Finnegan 2010; Chiou and Lin 2011).

### Interactions of Pi supply and auxin

The other major player in the control of RSA is auxin. The hormone has long been proposed to be a major regulator from the early pea root decapitation experiments of Wightman *et al.* (1980). More recent evidence of a role for auxin arose from *in vivo* studies using developmental mutants of *Arabidopsis* (Boerjan *et al.* 1995) or using factors that influence endogenous auxin transport (Reed *et al.* 1998). Thus the interaction of Pi status, changes in RSA and the role of auxin is worthy of record, and a number of studies, primarily in *Arabidopsis*, have implicated the hormone (López-Bucio *et al.* 2002; Al-Ghazi *et al.* 2003; Nacry *et al.* 2005; Jain *et al.* 2007; Perez-Torres *et al.* 2008; Miura *et al.* 2011). Of these, López-Bucio *et al.* (2002) used an array of auxin transport and signalling mutants of *Arabidopsis* and concluded that low P served to increase the sensitivity of the roots to respond to auxin in terms of inducing the signature changes in RSA. Using a similar panel of mutants, Nacry *et al.* (2005) concluded that changes in auxin distribution/transport and/or synthesis may underpin these changes such that in low P, an over-accumulation of auxin in the PR represses elongation but activates existing LR primordia and the emergence of the laterals, while a decrease in auxin in the elongation zone decreased the formation of new lateral primordia. In support of this, Miura *et al.* (2011) transformed mutants of the SUMO E3 ligase (SIZ1) of *Arabidopsis* that had an enhanced Pi response with *DR5p::uidA* and determined that the auxin accumulated earlier both in the PR tip (to decrease growth) and in the lateral primordia (to activate growth) in the *siz1* background when compared with wild type. Interestingly, Miura *et al.* (2005) had shown previously that SIZ1 interacts with PHR1, a global regulator of the systemic Pi responses (Bustos *et al.* 2010; Thibaud *et al.*, 2010), and the *siz1* mutant was shown to have elevated Pi levels. In terms of mechanism, Perez-Torres *et al.* (2008) looked at how this change of sensitivity may be mediated and concluded that Pi deprivation increases the expression of *TIR1* and the action of auxin then stimulates the degradation of the Aux/IAA repressors, thus sensitizing the tissue to auxin.

## Phosphate Supply and Ethylene Also Interact to Influence RSA

### How does ethylene influence root growth?

Prior to a discussion as to how low Pi supply and ethylene interact to influence RSA, it is pertinent to review how the hormone influences root growth in P-supplied roots. The involvement of ethylene as a regulator of root growth has been well established since the early experiments of Smith and Russell (1969) with roots of barley. More recent evidence with *Arabidopsis* includes perturbations that can regulate ethylene evolution, such as a mutation in the Cullin-RING ubiquitin ligase, CULLIN3, which leads to the stabilization and thus accumulation of 1-aminocyclopropane-1-carboxylate (ACC) synthase 5 (ACS5), and an up-regulation of ethylene biosynthesis. In these plants, repression of PR elongation is observed (Thomann *et al.* 2009). A general consensus thus exists whereby lower concentrations of the hormone can promote root extension, while higher concentrations are inhibitory for growth (Jackson *et al.* 1981; Jackson 1991), so underlining the biphasic action of ethylene in regulating many aspects of vegetative growth (Pierik *et al.* 2006).

The role of ethylene in regulating RSA, and how this is mediated *via* auxin, is also well established. In the PR of *Arabidopsis*, it is clear that ethylene is stimulating auxin biosynthesis in the root tip, where the higher concentration that ensues inhibits growth, and also a redistribution to the elongation zone (Ruzicka *et al.* 2007; Swarup *et al.* 2007; Strader *et al.* 2010). For the PR of *Arabidopsis*, some details as to the downstream mechanism of inhibition are emerging, which suggests that ethylene may work *via* auxin by increasing the cellular concentration of the hormone to a level that inhibits the auxin target H^+^-pumping ATPases so maintaining apolastic alkalinization. Ethylene may also increase ROS production that stimulates hydroxyproline-rich glycoprotein cross-linking which, acting in concert with alkaliniation, will serve to reduce the extensibility of the wall (Staal *et al.* 2011).

Ethylene also regulates the initiation of LR primordia and LR outgrowth, again controlled *via* auxin biosynthesis and distribution (Ivanchenko *et al.* 2008; Negi *et al.* 2008). In *Arabidopsis*, higher ethylene concentrations were observed to inhibit the ability of the pericycle to initiate new root primordia although the hormone did promote the emergence of existing primordia (Ivanchenko *et al.* 2008). Supporting evidence for the requirement for ethylene also arises from studies in which a mutant of *Arabidopsis* with a lesion in a member of the RING domain-containing ankyrin repeat subfamily of E3 ligases, XBAT32, resulted in ACS accumulation, an increase in ethylene biosynthesis and the production of fewer LRs in the *xbat32* background (Prasad *et al.* 2010). In terms of the mechanism of action of ethylene in LR formation in *Arabidopsis*, one proposed role for the hormone is to up-regulate the expression of *PIN3* and *PIN7* resulting in increased auxin transport and the lack of establishment of a sufficient local auxin concentration to initiate new LR primordia (Lewis *et al.* 2011). For a detailed account of the interaction between auxin and ethylene and the initiation and control of root growth and RSA, the reader is referred to the comprehensive review by Muday *et al.* (2012). However, of more pertinence to this review is the emerging view that P status regulates RSA architecture through documented changes in auxin biosynthesis and transport which is controlled ultimately *via* ethylene biosynthesis and/or signalling.

### The influence of P supply on ethylene biosynthesis and signalling

Given the pertinence of the links between auxin signalling, phosphate supply and a role for ethylene, there have been few documented studies where investigators have measured ethylene levels in P-starved roots, and few in general on the regulation of ethylene biosynthesis in P-starved or P-supplied roots. Notably, Tsuchisaka and Theologis (2004) examined the differential expression of *ACS* genes in P-supplied roots of *Arabidopsis*, while Gallie *et al.* (2009) examined the tissue-specific expression of both *ACS* and *ACC oxidase (ACO)* genes in the elongating P-supplied root of maize and concluded that ethylene production was limiting for root elongation, particularly the ACS isoform, *ZmACS6*. In terms of ethylene and altered P supply, Drew *et al.* (1989), using maize in hydroponic culture, excised apical segments of emerged adventitious roots and noted that ethyene evolution, ACC levels, and ACO and ACS activity decreased, leading to the conclusion that P-starvation-induced aerenchyma formation was not directed by ethylene. In careful experiments with bean, Borch *et al.* (1999) determined that P deficiency did not result in increased ethylene evolution at the root zone, although if the total root dry weight was compared, then an increase was noted in the low P treatment (to reflect the decreased total root mass). In tomato, low P treatment significantly increased ethylene production from the shoots, but decreased it in the roots, and while adventitious roots produced more ethylene, a significant decrease was noted in response to low P supply (Kim *et al.* 2008). In this study, the ethylene evolution values are expressed on a per fresh weight (FW) basis, which may accentuate the differences given that lower FW values were again recorded for both shoots and roots in the P-deprived treatments.

**Figure 1.**
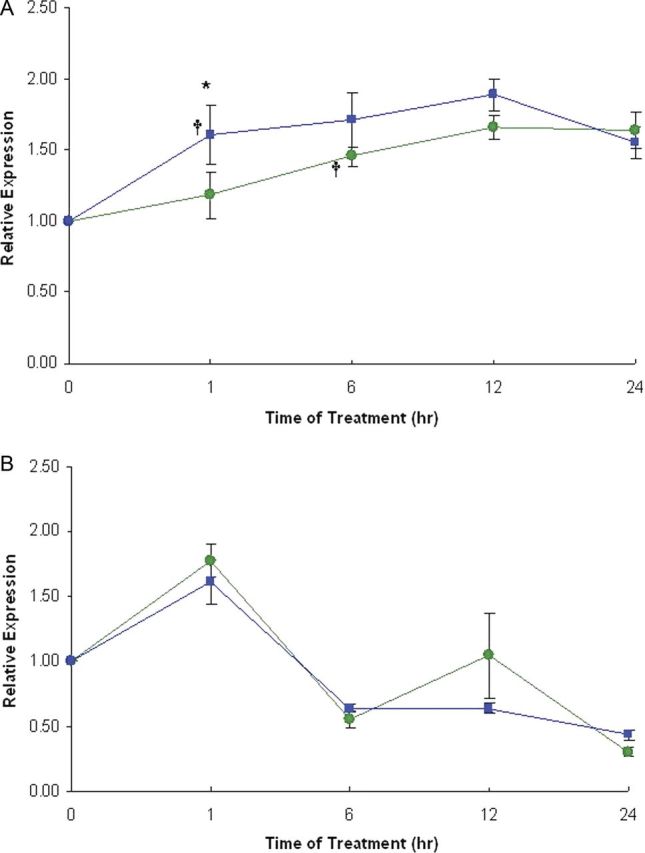
The multi-gene ACO family displays differential expression in roots in response to the low Pi treatment. The effects of P treatment [1 mM, P+ (-•-); 10 µM, P− (-▪-)] on the expression, as determined by qPCR, of *TR-ACO1* (A) and *TR-ACO2* (B) in the roots of wild-type white clover harvested at the time intervals indicated. The * indicates significant differences between treatments (*P*≤ 0.05); † indicates significant differences within a treatment from day 0.

Using another approach, large-scale transcriptome analyses can be used to screen for the up-regulation of the ethylene biosynthetic genes. Hernandez *et al.* (2007) detected the up-regulation of an *ACO* gene in bean that was confirmed using quantitative polymerase chain reaction (qPCR) expression analysis. This confirmed an earlier study in which gene cluster analysis across five legume species was used to extend the annotation of *P. vulgaris* genes where, again, an up-regulation of an *ACO* gene was observed (Graham *et al.* 2006). In their large-scale transcriptome study in *Arabidopsis*, Thibaud *et al.* (2010) also identified an *ACO* gene (*AtACO4*) as a member of a cadre of up-regulated genes that are locally induced, suggesting that an early response of plant roots to P deprivation is the induction of ACO (and possible stimulation of ethylene biosynthesis) irrespective of root Pi status. However, in another *Arabidopsis* transcriptome study, Chacon-Lopez *et al.* (2011) compared gene changes in the *low phosphorus insensitive 4* mutant background with wild type. The *lpi4* mutants do not show the switch to a determinate root growth in response to low Pi leading to an extended PR. Thus comparison between global changes in the mutant with wild type could identify genes that are enriched for response to Pi deprivation, such that genes that are down-regulated in the *lpi4* background should correspond to those that are up-regulated in low Pi-treated wild-type plants. However, one *ACO*-like transcript, which is distinct from *AtACO4* identified by Thibaud *et al.* (2010), was observed to be up-regulated in the mutant background in response to low P (Chacon-Lopez *et al.* 2011). Such observations suggest that the multi-gene *ACO* family displays differential expression in roots in response to the low Pi environmental cues. This is underlined in the roots of the pasture legume, white clover (*Trifolium repens*), where we have determined that one member of the *ACO* multi-gene family, *TR-ACO1*, responds to the low P treatment rapidly (by 1 h), while a second member of the family, *TR-ACO2*, does not respond in the same way over the same time frame (Fig. 1). Thus, as noted by Kim *et al.* (2008), any up-regulation of ethylene biosynthesis in response to low P is likely to be very tissue- and developmental-stage-dependent. While transcriptional studies can identify the up-regulation of the ethylene biosynthetic genes, it is important to know in which tissues the genes are being expressed and at which stage during the Pi-deprivation response.

### The influence of low-P-induced ethylene on RSA

The key impetus for the investigation of changes in ethylene biosynthesis in response to P supply has been to characterize how the hormone does influence RSA in a P-starved tissue. Historically, approaches have progressed from the application of the hormone to the roots (often as added ACC), to the use of both ethylene biosynthesis and action inhibitors through to mutant backgrounds that are compromised in either ethylene signalling or P response. In earlier work, He *et al.* (1992) showed in maize that low P did not induce a perceptible increase in ethylene production, but it did result in sensitizing the tissues to exogenously applied ethylene in terms of the induction of aerenchyma formation. In bean, Borch *et al.* (1999) showed that inhibiting ethylene biosynthesis with aminoethoxyglycine (AVG) abolished the documented low-P-induced changes to RSA. However, these workers also determined that the response to ethylene was, more correctly, determined by tissue and P supply such that in the PR, ethylene promotes growth in low P and inhibits it in sufficient P. Likewise, in the determination of LR density, ethylene decreases density in low P (Borch *et al.* 1999). These results in bean have been summarized by Lynch and Brown (2001) in which they proposed that P availability affects ethylene responsiveness that operates conversely in PR growth and LR density. Thus, in bean, an increase in measured ethylene concentration growth in low P stimulated the growth of the PR and decreased LR frequency.

In *Arabidopsis* too, Ma *et al.* (2003) measured the rate of PR elongation and concluded that while the rate is decreased in low P, ethylene is important for the maintenance of root elongation and further that the roots are more sensitive to ethylene in the low P media [as determined by the use of 1-methylcyclopropene (1-MCP) and AVG]. These studies highlight that ethylene production *per se* is one aspect of the role of the hormone, but sensitivity is equally important (and may be the ultimate regulator). In tomato and Petunia, the use of ethylene-insensitive mutants and the measurement of ethylene production allowed Kim *et al.* (2008) to conclude that, in these species at least, low P did not cause an increase in ethylene production, and so the changes in RSA measured were orchestrated *via* changes in sensitivity. Likewise, in the pasture legume white clover, Dinh *et al.* (2012) showed that ACC added to roots maintained in low P induced a ‘super-stimulation’ of LR number and mean LR length when compared with the stimulation induced by the low P treatment alone.

To examine the mechanism by which ethylene may alter root growth, albeit *via* an increased sensitivity to the hormone, Chacon-Lopez *et al.* (2011) examined the P-induced cessation of PR growth in *Arabidopsis* utilizing a QC marker. They noted that the previously observed consumption of the root meristem in the P-deficient treatment was abolished if either AVG or silver ions (Ag^2+^) were added. Further, if ACC was added to the P-sufficient media, consumption of the meristem was again observed although this did not proceed to completion, suggesting that ethylene does not act alone. However, the addition of jasmonic acid (JA) and ethylene did promote complete meristem consumption, implying that the rapid changes in RSA may have similarities to broad stress-associated responses. Finally, Yu *et al.* (2012) characterized the *hypersensitive to Pi starvation 4 (hps4)* mutant of *Arabidopsis*, a background that displays an exaggerated response in terms of RSA, and found it to be allelic to *SABRE*, a previously characterized gene that regulates cell expansion. A link with ethylene signalling in the P response was established when it was shown that added Ag repressed the observed increase in severity of phenotype in low P. Interestingly, these workers also observed that the mutant accumulated a higher auxin content in the apical 5 mm root tips as well as displaying an increased expression of auxin-biosynthesis-associated genes in harvested seedlings, leading the mechanism back to the phenomenon described in this review of ethylene-induced changes in auxin biosynthesis and signalling.

## Ethylene Also Regulates Other Signature Responses to P Supply in Addition to RSA

It is now accepted that the influence of P-induced ethylene biosynthesis or signalling and changes in RSA is mediated, at one level, *via* the ethylene interactions with auxin. However, auxin does not induce the expression of the phosphate-starvation inducible (PSI) genes, and so the focus of research has shifted more recently to examining the possible other roles of ethylene in terms of inducing the PSI genes and influencing activity.

Lei *et al.* (2011) characterized the *hypersensitive to phosphate deprivation 2 (hsp2)* mutant of *Arabidopsis* that displays an exaggerated response to Pi deficiency including increased inhibition of PR growth and a more marked induction of *AtPT2* expression. The mutation is allelic to the *CTR1* gene, suggesting that the phenotype was due to increased ethylene biosynthesis. Therefore to look at the role of ethylene more directly in terms of the regulation of PSI gene expression, *AtPT2* expression was characterized in the *etr1-1* and *ein2-5* mutant backgrounds and found not to be increased in response to low P, while induction of *AtPT2* expression was observed in the *eto1-1* background (Lei *et al.* 2011). To more directly examine the role of ethylene in the induction of *AtPT2*, these workers also showed that added ACC could induce *AtPT2* expression in low-P-treated plants, but not in the P-sufficient treatment, so underlining that low P sensitizes the plant to respond to ethylene. For the acid phosphatase (Apase) enzyme, root surface activity was highest in the *hps2* mutant, followed by wild type with very little induction in the *ein2-5* background in response to low P. Using another mutant of *Arabidopsis*, Wang *et al.* (2012) characterized *hypersensitive to phosphate starvation 3* (*hps3*) and showed that it was a mutation in the *ETO1* gene, thus giving an ethylene ‘on’ phenotype. In common with other studies, these workers showed that treatment of the mutant with either AVG or Ag^2+^ abolished the exaggerated root growth phenotype observed in response to low P treatment. Further, they also showed that there was an induction of root surface Apase (but not total Apase) and an induction of several PSI genes examined in the mutant that could also be abolished if treated with AVG and Ag^2+^.

In other species, Li *et al.* (2011) showed that for *Medicago falcata*, transfer of plants from high to low P medium caused a decrease in Pi content in the roots that could be abolished if the plants were treated with AVG or Co^2+^ (to inhibit ACO activity). Also, if plants that are maintained in high P are treated directly with ACC, then the Pi content of the roots decreases, an observation that was supported by examination of the expression of a higher-affinity transporter *MfPT1* that was up-regulated by Pi deficiency and reversed by AVG and Co^2+^. In common with *Arabidopsis*, surface Apase was stimulated in roots of P-sufficient plants treated with ACC, while the induction of activity in the P-deficient medium was abolished by AVG treatment. Taken together, these results do support the contention that ethylene may have pleiotropic effects in terms of mediating the responses of plant roots to P deficiency, and it is likely that further roles will be identified in the future.

## Conclusions

**Figure 2.**
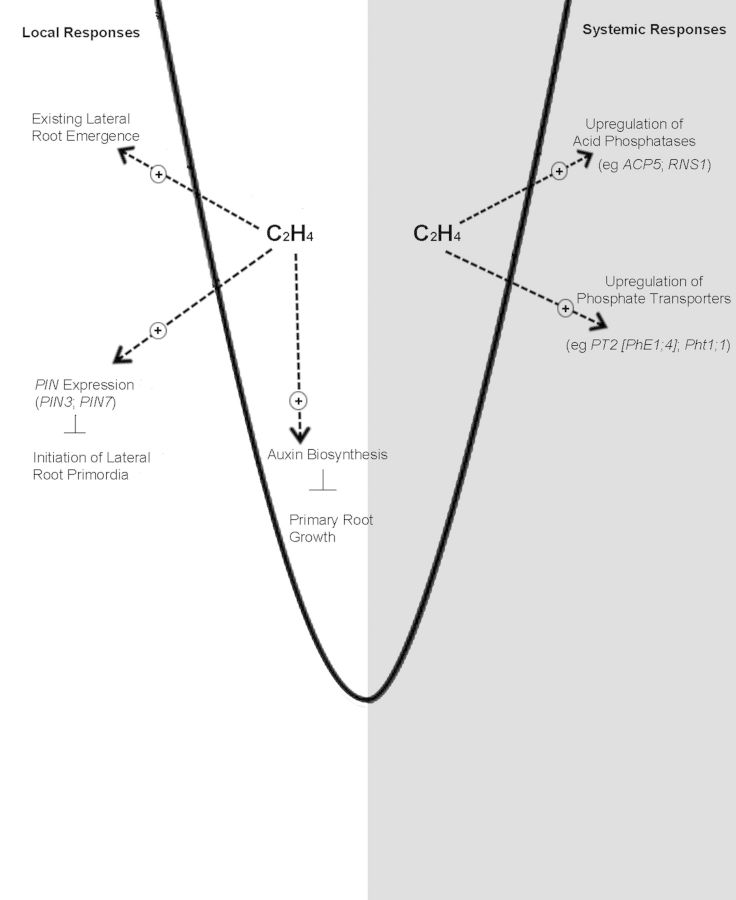
Overview of the proposed dual role of ethylene in *Arabidopsis* in response to low Pi supply. The summary is predicated on the key observation that the occurrence of the hormone is a local response to changes in external Pi and involves increased biosynthesis (e.g. up-regulation of *AtACO4*; Thibaud *et al.* 2010) and/or increased sensitivity (e.g. up-regulation of *AtERF* genes; Thibaud *et al.* 2010). These local changes in ethylene concentration/sensitivity then interact with auxin to mediate changes to RSA. Any ensuing changes in P status of the leaves evoke a second tier of changes, the systemic responses, whereby the hormone induces, but independent of auxin, a series of key signature changes to P depletion including the up-regulation of acid phosphatases and phosphate transporters.

Advances in our understanding of the responses of plants to P deficiency and the interactions with ethylene lead to the scenario of a multi-faceted role for the hormone, inviting the possibility of distinct cues for ethylene biosynthesis and changes in sensitivity. In contrast is the realization and acceptance that changes in external Pi can act both as a signal for the root and as an essential macro-element for metabolism. It is apparent that one of the signalling roles of Pi is to induce the transcription of the ethylene biosynthetic genes, and for ACO at least, this can occur very rapidly. Evidence for any coarse changes in the production of the hormone is not consistent, as some studies have reported an increase in evolution, while others a decrease. It is more likely that any changes are cell- and/or tissue-specific with concurrent changes in sensitivity also an important regulator. The role of these changes in biosynthesis and sensitivity may be to evoke a series of protectant-type responses (and it is significant that another stress-associated hormone, JA, is also required), as well as playing a key role in the signature changes in RSA as the roots seek out P-sufficient patches in soil. For these, the interaction with auxin is paramount (summarized in Fig. 2).

A second series of events, the systemic responses, are denoted by changes in the Pi-deprivation metabolic responses caused by complex root–shoot–root signalling and are reflective of changes in the endogenous Pi levels. More recently, ethylene, in a mechanism apparently independent of auxin, has been shown to also induce many of these changes (summarized in Fig. 2), but it is not yet clear whether a distinct series of systemic cues induce ethylene production and/or changes in ethylene sensitivity (Nagarajan and Smith 2012). Thus it is important to look further at the nature of the transcriptional cues that regulate ethylene biosynthesis and perception. As the hormone does play such a multi-faceted role in the response to P deficiency, any revelations of such control must reveal further avenues to improve phosphate use efficiency.

## Sources of Funding

The research reported in this article was funded by the New Zealand Foundation for Science, Research and Technology and the Massey University Research Fund.

## Contributions by the Authors

M.T.M. drafted the article and M.R., P.D. and S.L. commented on the draft.

## Conflict of Interest Statement

None declared.
